# Quality of antibody responses by adults and young children to 13-valent pneumococcal conjugate vaccination and *Streptococcus pneumoniae* colonisation

**DOI:** 10.1016/j.vaccine.2022.09.069

**Published:** 2022-11-28

**Authors:** Asia-Sophia Wolf, Elena Mitsi, Scott Jones, Simon P. Jochems, Lucy Roalfe, Deus Thindwa, James E. Meiring, Jacquline Msefula, Farouck Bonomali, Tikhala Makhaza Jere, Maurice Mbewe, Andrea M. Collins, Stephen B. Gordon, Melita A. Gordon, Daniela M. Ferreira, Neil French, David Goldblatt, Robert S. Heyderman, Todd D. Swarthout

**Affiliations:** a NIHR Global Health Mucosal Pathogens Research Unit, Division of Infection and Immunity, University College London, London, UK; b Department of Clinical Sciences, Liverpool School of Tropical Medicine, Liverpool, UK; c Great Ormond Street Institute of Child Health, University College London, London, UK; d Malawi-Liverpool-Wellcome Programme, Blantyre, Malawi; e Department of Infectious Disease Epidemiology, London School of Hygiene and Tropical Medicine, London, UK; f Department of Infection, Immunity and Cardiovascular Disease, University of Sheffield, UK; g Liverpool University Hospitals Foundation Trust, Liverpool, UK; h Kamuzu University of Health Sciences, Blantyre, Malawi; i Institute of Infection, Veterinary and Ecological Sciences, University of Liverpool, Liverpool, UK

**Keywords:** Pneumococcal conjugate vaccine, Carriage, Colonisation, Pneumococcus, Opsonophagocytosis, Avidity, PCV

## Abstract

Childhood pneumococcal conjugate vaccine (PCV) protects against invasive pneumococcal disease caused by vaccine-serotype (VT) *Streptococcus pneumoniae* by generating opsonophagocytic anti-capsular antibodies, but how vaccination protects against and reduces VT carriage is less well understood. Using serological samples from PCV-vaccinated Malawian individuals and a UK human challenge model, we explored whether antibody quality (IgG subclass, opsonophagocytic killing, and avidity) is associated with protection from carriage. Following experimental challenge of adults with *S. pneumoniae* serotype 6B, 3/21 PCV13-vaccinees were colonised with pneumococcus compared to 12/24 hepatitis A-vaccinated controls; PCV13-vaccination induced serotype-specific IgG, IgG1, and IgG2, and strong opsonophagocytic responses. However, there was no clear relationship between antibody quality and protection from carriage or carriage intensity after vaccination. Similarly, among PCV13-vaccinated Malawian infants there was no relationship between serotype-specific antibody titre or quality and carriage through exposure to circulating serotypes. Although opsonophagocytic responses were low in infants, antibody titre and avidity to circulating serotypes 19F and 6A were maintained or increased with age. These data suggest a complex relationship between antibody-mediated immunity and pneumococcal carriage, and that PCV13-driven antibody quality may mature with age and exposure.

## Highlights

• PCV13 reduces colonisation after experimental *S. pneumoniae* challenge in adults.
• Vaccinated adults had strong serotype-specific functional antibody responses.
• Unvaccinated adults with high avidity antibodies were less likely to be colonised.
• Residual carriage of vaccine serotypes maintains IgG titres in vaccinated children.
• Vaccinated children maintain serotype-specific avidity without antigen re-exposure.

## 1 Background

*Streptococcus pneumoniae* is commonly carried as a commensal bacteria in the nasopharynx but can cause life-threatening disease, including pneumonia, meningitis and sepsis, particularly in children aged under 5 years, the immunocompromised, and older people [1]. Pneumococcal capsular polysaccharide (CPS) conjugate vaccines (PCV), which contain and protect against the most common disease-causing serotypes, are widely given as part of routine infant vaccination programmes across the world [2]. PCVs have effectively reduced invasive pneumococcal disease caused by vaccine serotypes (VT-IPD) [3], [4], [5], [6] and in high-income settings have reduced colonisation sufficiently to provide considerable herd immunity [3], [7]. However, VT-IPD and colonisation by vaccine serotypes in children within low- and middle-income countries remains higher than in high-income countries even with excellent (>90%) vaccine uptake [8], [9], [10], [11], [12], [13], [14]. To further improve PCV impact in high pneumococcal carriage settings, it is therefore important to understand how these vaccines control acquisition of carriage.

Pneumococcal CPS induces a B cell-mediated antibody response [15], [16]. Although the protective titre varies by serotype, serum IgG titres of >0.35 μg/ml are considered protective against IPD [17], [18], [19], [20]. However, although circulating IgG may correlate with protection against colonisation, the required antibody titres are less well defined and are likely to be several times higher than the correlate of protection (CoP) for IPD [14]. Voysey *et al* have proposed serotype-specific protective antibody levels in children based on associations between serotype-specific antibody titres and seroincidence that range from 0.5 to 2.54 μg/ml [9]. Additionally, CoPs against colonisation were estimated to be on average twice as high in high disease and carriage burden countries than in low burden countries [9], [14]. These serotype-specific CoPs appear generally applicable to Malawian children [14] but do not fully explain vaccine-mediated control of colonisation. Other immune parameters including antibody quality, polysaccharide-specific memory B cells, and antibody-mediated agglutination of bacteria at the mucosal surface have therefore been suggested as protective against carriage acquisition [21], [22], [23].

Although both IgG1 and IgG2 contribute to complement-mediated killing of bacteria [24], IgG1 mediates opsonophagocytosis through interactions with cellular Fc receptors [25]. IgG2 effector functions are less clear, but it is the predominant subclass produced in response to polysaccharides, including pneumococcal CPS [26], [27]. After vaccination and natural exposure, the relative proportions of IgG1 and IgG2 vary by age, but antibody responses in children after PCV vaccination are skewed more to IgG1 production compared to adults [26], [28]. Opsonophagocytic killing (OPK) activity increases after PCV vaccination [29] and has been used to assess vaccine-mediated protection against IPD [30], but does not consistently correlate with protection against pneumococcal colonisation [20]. Antibody avidity has also been used as a measure of antibody quality in studies of pneumococcal vaccines [21], [31], [32], [33] and other encapsulated bacteria [34] but has not been widely assessed in relation to pneumococcal carriage.

We have therefore explored whether, in addition to total antibody titre, the quality of the antibody response to vaccination (IgG subclass, OPK activity and avidity) are determinants of pneumococcal colonisation. To test this, we analysed serum from UK adults who had been immunised with either one dose of PCV13 or a control vaccine prior to Experimental Human Pneumococcal Challenge (EHPC) [7]. To further evaluate these findings in a vaccinated and naturally exposed population, we have used population-based carriage surveillance data [11] and a large scale serosurvey [14], [35] of children in Blantyre, Malawi.

## 2 Methods

**Experimental Human Pneumococcal Challenge model**: Venous blood samples were taken from adults, aged 18–50 years, at baseline prior to vaccination with either PCV13 (Prevnar, Pfizer) or a control inactivated Hepatitis A vaccine (HepA; Avaxim, Sanofi Pasteur MSD). Blood samples were collected 4–5 weeks post-vaccination prior to intranasal inoculation with live penicillin-sensitive serotype 6B *S. pneumoniae* (BHN418) (80,000 CFUs per nostril) [36]. A third blood sample was taken 21 days after challenge alongside nasal washes to determine colonisation with 6B pneumococcus. Colonisation density was measured from nasal wash samples at 2, 7, 14 and 21 days after challenge and integrated into a single value representing intensity of colonisation (area under the curve, AUC) as previously described [7]; briefly, nasal wash samples were collected by washing each naris with 10 ml 0.9% saline solution, centrifuged to obtain a bacterial pellet, and plated out at serial dilutions on Columbia Horse Blood Agar containing 4 µg/ml gentamicin (Sigma) to quantify colonisation density. Pneumococcal phenotype was identified after incubation for 24 h at 37°C with 5% CO_2_; serotype was determined by latex agglutination. Individuals with detectable experimental pneumococci at any time point post-challenge were considered experimentally colonised. All experimentally colonised individuals who did not have two consecutive culture-negative nasal washes received amoxicillin for 3 days at the end of the study to ensure clearance of colonisation. Some participants reported symptoms after vaccination and challenge as described in [7] but none became ill or required further treatment. AUC values were calculated using the trapezoidal rule based on measured pneumococcal colonisation density at the four sampling time points.

**Serum collection**: UK adult serum samples were obtained from a PCV13 double-blind randomized controlled trial with an Experimental Human Pneumococcal Challenge (EHPC) as described above [7]. Malawi serum samples were randomly selected from a biobank generated during a serosurvey conducted between 14th December 2016 and 12th April 2018 as part of the STRATAA (Strategic Typhoid Alliance across Africa and Asia) study in Blantyre, Malawi [35]. Blantyre is located in southern Malawi and has an urban population of approximately 1.3 million. The analysis using STRATAA samples included samples from age-stratified healthy children under 5 years of age [35]. All child participants were confirmed to have received the age-appropriate doses of routine infant PCV13 (given at ages 6, 10 and 14 weeks) as documented in the child health passport (patient retained medical record). Samples for this analysis were chosen from recently vaccinated younger children aged 3–9 months and older children aged 24–59 months, representing antibodies at both the post-vaccination peak and post-vaccination waning responses respectively. In 2011, Malawi adopted the WHO-recommended Option B+, whereby all HIV-infected pregnant or breastfeeding women commence lifelong antiretroviral therapy regardless of clinical or immunological stage, dramatically reducing mother-to-child transmission of HIV, and HIV prevalence is therefore extremely low in these age groups.

**Antibody titres**: Serum IgG titres were measured by direct ELISA as described in the WHO protocol for quantitation of *S. pneumoniae* serotype-specific IgG [37]. Plates were coated with purified capsular polysaccharide (CPS) from serotypes 6B, 19F, 6A or 7F (ATCC) at 5 μg/ml overnight at 4°C. Serum was absorbed with 5 μg/ml cell wall polysaccharide (CWPS Multi, SSI-Diagnostica) prior to incubation with CPS-coated plates. Serum was incubated on plates for 2 h at room temperature before incubation for another 2 h at room temperature with goat anti-human IgG conjugated to alkaline phosphatase (Southern Biotech, cat. no. 2040–04) or horseradish peroxidase (Sigma, cat. no. AP112P). Plates were developed with *p*-nitrophenyl phosphate (Sigma) or 3,3′,5,5′-Tetramethylbenzidine (TMB) solution (Thermo Scientific). Optical density was measured at 405 nm or 450 nm. Reference serum 007sp (NIBSC) was run on each plate and used to quantify antibody titres for unknown sera.

IgG1 and IgG2 titres were measured by indirect ELISA using primary anti-IgG1 or anti-IgG2 (Sigma, cat. no. I5385 and I9513) as above; after incubating CWPS-absorbed serum on coated plates, samples were incubated with primary mouse anti-human IgG1 or mouse anti-human IgG2 antibodies for 2 h at room temperature, washed, and further incubated with rabbit anti-mouse IgG conjugated to peroxidase (Sigma) before development with TMB. Reference serum 007sp was used to quantify IgG1 and IgG2 titres in samples based on calculations by Jones *et al* [38].

**Multiplex opsonophagocytosis assay (MOPA)**: Opsonophagocytosis of serotypes 6B, 19F and 6A was measured at the UCL Institute of Child Health as described in the UAB-MOPA protocol [39]. Briefly, cassettes of bacterial serotypes, each resistant to a different antibiotic, were incubated in a 96-well plate with serial dilutions of human serum before further incubation with HL-60 cells and baby rabbit complement. Wells were plated out onto agar with an agar overlay containing an antibiotic before overnight incubation, allowing only the antibiotic-resistant strain to grow. Bacterial colonies were quantified by a plate reader. OPA titres were expressed as the opsonic index (OI) where diluted serum kills 50% of bacteria of the specific serotype.

**Antibody avidity**: IgG avidity was measured by titrating sodium thiocyanate (NaSCN) into a known concentration of serum in an ELISA-based assay [34], [40], [41]. Microtitre plates coated with CPS from 6B, 19F, 6A or 7F for a standard ELISA as above were incubated for 2 h at room temperature with a fixed concentration of serum in replicate wells to give a final optical density reading of ∼1.0 (determined previously by ELISA). Concentrations of NaSCN (Sigma) ranging from 0 to 4 M were added to the wells and incubated for 15 min at room temperature before thorough washing. Goat anti-human IgG antibody was then added as for an ELISA and the remaining steps followed as described above. The avidity index (AI) of each sample is expressed as the molar concentration of NaSCN at which 50% of serum antibodies bound to the plate were disrupted. Reference serum 007sp was run on each plate as a control and the coefficient of variation per plate was <15%.

**Statistical analysis**: Statistical analyses of these data was carried out in GraphPad Prism v8.4.1. Statistical tests were performed using linear regression, Wilcoxon signed rank tests and Mann-Whitney tests, indicated for each figure. P-values of <0.05 were considered significant.

**Ethical approval**: The STRATAA study was approved by the Oxford Tropical Research Ethics committee (Ref: 39–15) and the Malawi National Health Sciences Research Committee (Ref: 15/5/1599). For the EHPC samples, ethical approval was obtained from the UK National Health Service Research Ethics Committee (Ref: 12/NW/0873).

## 3 Results

### 3.1 The relationship between IgG titres to serotype 6B following PCV13 or HepA immunisation and experimental pneumococcal colonisation

**Fig. 1:**
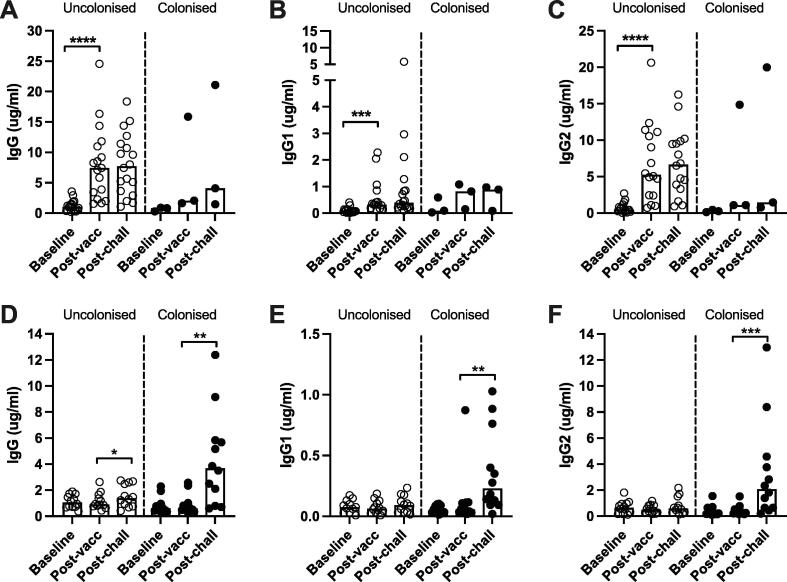
Antibody titres to *S. pneumoniae* serotype 6B in PCV13-vaccinated adults and HepA-vaccinated controls from EHPC. Bar graphs indicate IgG, IgG1 or IgG2 titres for PCV13-vaccinated (n = 21) (A-C) and HepA-vaccinated (n = 24) (D-F) individuals at three sample collection points. Samples are divided by colonisation outcome after experimental challenge with serotype 6B as part of the EHPC model (uncolonised shown by empty circles, colonised by filled circles). Statistical analysis was performed using Wilcoxon signed rank tests, bars indicate median values. * p <0.05, ** p <0.01, *** p <0.001, **** p <0.0001.

We measured serotype 6B-specific IgG, IgG1 and IgG2 titres in 21 PCV13-vaccinated adults and 24 HepA-vaccinated controls at baseline, post-vaccination, and post-challenge with *S. pneumoniae* serotype 6B (Fig. 1) [7], [23]. Baseline IgG titres were similar in the PCV13-vaccinated and HepA-vaccinated control groups. Following experimental challenge with *S. pneumoniae* serotype 6B in the original study, only 10.4% (5/48) of PCV13-vaccinated individuals became carriage positive, in contrast to 47.9% (23/48) of the HepA controls [7]. In this study, vaccination with PCV13 induced an IgG titre of >1.0 µg/ml in all (21/21) individuals (Fig. 1A); all antibody responses against serotype 6B are summarised in Table 1. Although vaccination with PCV13 stimulated IgG1 production (Fig. 1B), this accounted for only a small proportion of the total IgG response, and IgG2 responses were strongly induced by PCV13 vaccination (Fig. 1C). Within the PCV-vaccinated group, there were no apparent differences between the post-challenge IgG, IgG1 or IgG2 titres of individuals who did and did not become colonised; however, the number of participants with carriage was low, precluding a robust statistical comparison. In HepA-vaccinated controls, challenge induced an increase in serotype-specific 6B IgG titres, predominantly in individuals where experimental colonisation was established (Fig. 1D). This increase was a mix of IgG1 (Fig. 1E) and IgG2 (Fig. 1F), but the level of IgG1/IgG2 production varied by individual and did not correlate (data not shown). There were no statistically significant differences between baseline IgG titres in control group participants who became colonised after challenge and those who did not.

**Table 1:** Geometric mean values and confidence intervals for adult EHPC data (serotype 6B).

		**Baseline (geometric mean)**	**95% CI**	**Post-vaccination (geometric mean)**	**95% CI**	**Post-challenge (geometric mean)**	**95% CI**
**PCV13**							
Uncolonised	IgG (ug/ml)	0.89	0.58–1.38	6.1	3.96–9.39	6.23	4.14–9.36
	IgG1 (ug/ml)	0.068	0.043–2.52	0.41	0.25–0.67	0.52	0.30–0.88
	IgG2 (ug/ml)	0.45	0.23–0.85	4.73	2.79–8.04	5.26	3.30–8.37
	Opsonic index	183	93–357	8668	3760–19985	3463	1284–9341
	Avidity index (M)	1.31	0.99–1.72	0.61	0.37–1.02	0.66	0.41–1.05
Colonised	IgG (ug/ml)	0.59	0.13–2.74	3.78	0.17–83.90	5.02	0.18–142.2
	IgG1 (ug/ml)	0.12	0.003–5.01	0.51	0.04–7.21	0.42	0.01–12.66
	IgG2 (ug/ml)	0.26	0.06–1.02	2.6	0.06–110.9	2.81	0.04–206.6
	Opsonic index	271	0–333964	2360	29–193214	1735	250–12060
	Avidity index (M)	1.28	0.51–3.20	0.9	0.24–3.43	1.07	0.31–3.74
**HepA**							
Uncolonised	IgG (ug/ml)	1.02	0.75–1.39	1.04	0.76–1.43	1.34	0.91–1.99
	IgG1 (ug/ml)	0.06	0.03–0.12	0.06	0.03–0.10	0.07	0.04–0.13
	IgG2 (ug/ml)	0.52	0.30–0.89	0.46	0.31–0.69	0.57	0.33–0.99
	Opsonic index	557	68–4531	908	455–1810	164	16–1669
	Avidity index (M)	1.58	1.28–1.94	1.51	1.25–1.82	1.68	1.41–2.00
Colonised	IgG (ug/ml)	0.66	0.44–1.00	0.69	0.44–1.07	2.96	1.57–5.62
	IgG1 (ug/ml)	0.05	0.03–0.07	0.07	0.04–0.13	0.22	0.11–0.46
	IgG2 (ug/ml)	0.3	0.18–0.49	0.33	0.21–0.52	1.8	0.81–3.96
	Opsonic index	339	17–6772	215	20–2356	411	88–1929
	Avidity index (M)	0.79	0.64–0.98	0.87	0.63–1.17	0.56	0.36–0.86

### 3.2 Opsonophagocytic killing against serotype 6B among PCV13- or HepA-immunised adults and the relationship to experimental pneumococcal colonisation

**Fig. 2:**
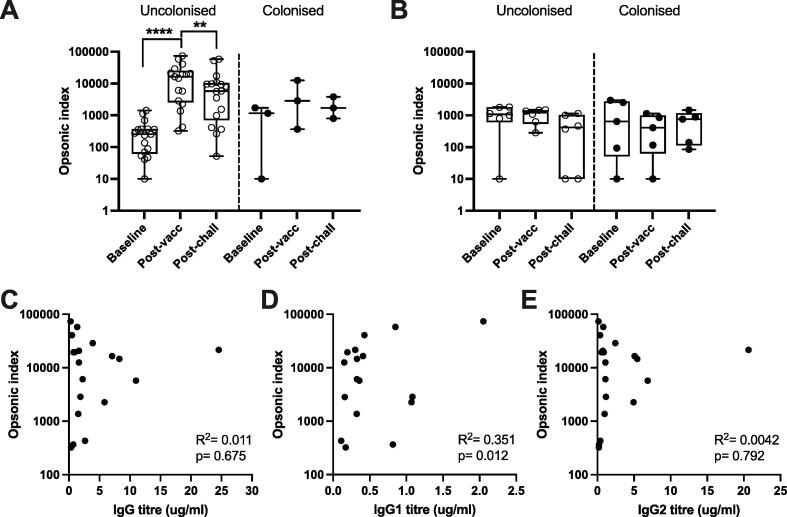
Opsonophagocytic killing responses to *S. pneumoniae* serotype 6B in PCV- and HepA-vaccinated adults. OPK by OPA in adult HepA-vaccinated (n = 11) (A) and PCV13-vaccinated (n = 20) (B) individuals. Data are split by colonisation outcome after experimental challenge with serotype 6B as part of the EHPC model. Statistical analysis was performed using Wilcoxon signed rank tests. Opsonic indices (OIs) from PCV13-vaccinated individuals post-vaccination were plotted against IgG (C), IgG1 (D) and IgG2 (E) titres. The R^2^ and p values indicate analysis via linear regression. * p <0.05, ** p <0.01, *** p <0.001, **** p <0.0001.

Among all individuals, there were no clear differences between the OPK responses of individuals who did or did not become colonised after challenge (Fig. 2 and Table 1). OPK responses remained low in the HepA-vaccinated group at all time points (Fig. 2A) (geometric mean OI at baseline: 293 [40–2135]; post-vaccination: 382 [91–1598]; post-challenge: 164 [27–985]). In the PCV-vaccinated group, OPK responses against serotype 6B were low at baseline for most individuals and were strongly enhanced one month following PCV13 vaccination (Fig. 2B) (geometric mean OI for all PCV-vaccinees at baseline: 154 [95% CI 59–404]; post-vaccination: 7132, [3268–15565]). The OPK response had significantly decreased by 7–8 weeks post PCV13 vaccination (geometric mean OI post-challenge: 3122 [1340–7273]). Challenge with 6B bacteria, regardless of colonisation status, did not boost OPK responses. After PCV vaccination, OPK responses modestly correlated with IgG1 titres (Fig. 2D) (R^2^ = 0.351, p = 0.012) but not IgG or IgG2 titres (Fig. 2C and 2E) (R^2^ = 0.011 and 0.0042, p = 0.675 and 0.792 respectively).

### 3.3 Antibody avidity against serotype 6B among PCV13- and HepA-immunised adults and the relationship to experimental pneumococcal colonisation

**Fig. 3:**
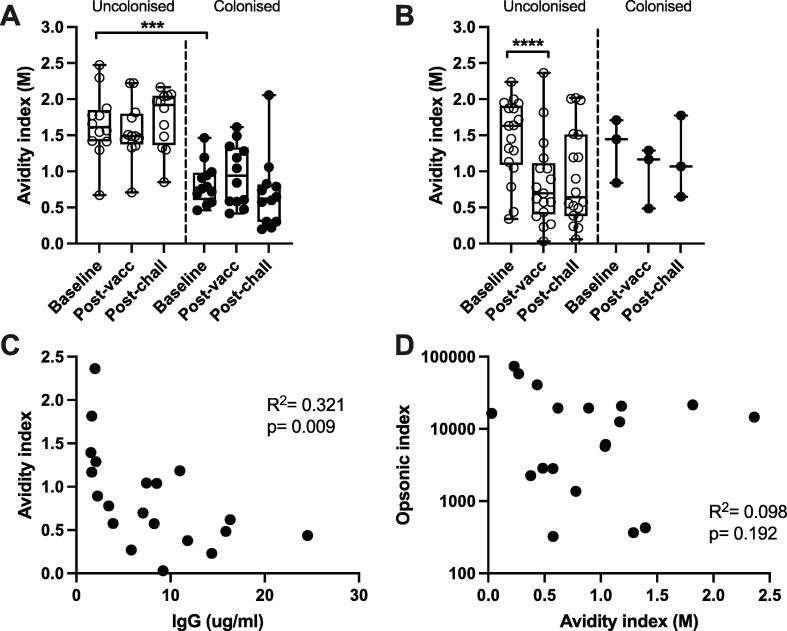
Antibody avidity to *S. pneumoniae* serotype 6B in HepA- and PCV13-vaccinated adults. Antibody avidity measuring IgG avidity by thiocyanate chaotropic disruption is given as avidity index (AI) in molar units of NaSCN. Data are divided by HepA-vaccinated ‘naturally exposed’ individuals (n = 24) (A) and PCV13-vaccinated individuals (n = 21) (B) and split by colonisation outcome after experimental challenge with serotype 6B as part of the EHPC protocol. Box-and-whisker plots indicate medians, IQR and min/max values; statistical comparison of baselines (A) by Mann-Whitney unpaired *t* test and baseline vs post-vaccination timepoints (B) by Wilcoxon signed rank test. Avidity indices from the post-vaccination samples were plotted against IgG titres (C) and opsonic indices (D) and analysed by linear regression for correlation. R^2^ values are indicated on the plots. ** p <0.01, *** p <0.001, **** p <0.0001.

Baseline 6B-specific IgG avidity was similar in both PCV13 and HepA vaccinated individuals (Fig. 3 and Table 1). However, participants in the control group with higher avidity antibodies at baseline were significantly less likely to become colonised upon challenge than individuals from the control group with lower avidity antibodies (p = 0.0002; geometric mean AI (GMAI) in uncolonised vs colonised individuals was 1.58 vs 0.79, 95% CI 1.28–1.94 vs 0.64–0.98) (Fig. 3A). By contrast, in PCV13 vaccinated individuals neither baseline avidity nor post-vaccination avidity correlated with colonisation upon challenge (Fig. 3B). Indeed, after vaccination and prior to challenge, the mean avidity decreased (p < 0.0001; GMAI in uncolonised individuals at baseline vs post-vaccination was 1.31 vs 0.61, 95% CI 0.99–1.72 vs 0.37–1.02). A similar decrease in avidity after vaccination was seen in 19F-specific antibodies (Supplementary Fig. 1). We also found that post-vaccination avidity negatively correlated with IgG concentration (Fig. 3C) (R^2^ = 0.321, p = 0.009). Avidity and OPK activity did not correlate after vaccination (Fig. 3D) (R^2^ = 0.098, p = 0.192) or at the baseline or post-challenge time points (data not shown).

### 3.4 Antibody profiles induced by natural exposure in adults and intensity of experimental colonisation with serotype 6B

**Fig. 4:**
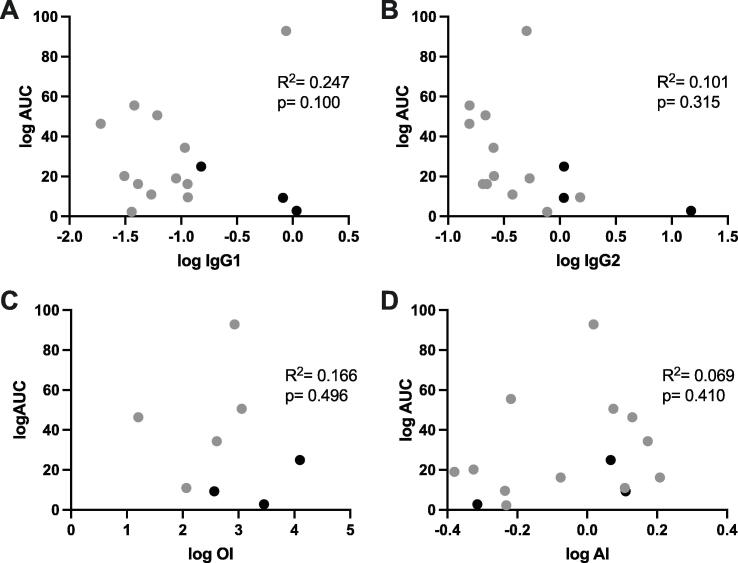
Relationship between intensity of colonisation and immunity to *S. pneumoniae* serotype 6B amongst PCV13-vaccinated and unvaccinated adults. Linear correlations of intensity of colonisation (AUC) against log-transformed IgG1 (A), IgG2 (B), OPK (C) and avidity (D) at the post-vaccination time point (pre-challenge). Grey circles represent unvaccinated individuals (HepA-vaccinated controls). R^2^ and p values shown refer to the linear correlation for unvaccinated controls only, excluding the three PCV13-vaccinated individuals (shown by black circles for reference). All p values are not significant.

There were too few carriage-positive PCV13-vaccinated individuals to determine which vaccine-generated antibody characteristics affected intensity of colonisation (AUC). However, in the HepA-vaccinated individuals we assessed whether anti-capsular antibodies, presumably induced through natural exposure prior to 6B challenge (i.e. the post-vaccination time point), had an effect on the intensity of colonisation (Fig. 4). However, none of the antibody characteristics we measured (IgG1, IgG2, OPK and avidity, Fig. 4A-D respectively, IgG not shown) showed significant correlation with colonisation intensity (IgG, R^2^ = 0.018, p = 0.676; IgG1, R^2^ = 0.247, p = 0.100; IgG2, R^2^ = 0.101, p = 0.315; OPK, R^2^ = 0.166, p = 0.496; avidity, R^2^ = 0.069, p = 0.410).

### 3.5 Antibody profiles against serotypes 19F, 6A and 7F in Malawian children after PCV13 immunisation and subsequent natural exposure to *S. pneumoniae*.

**Fig. 5:**
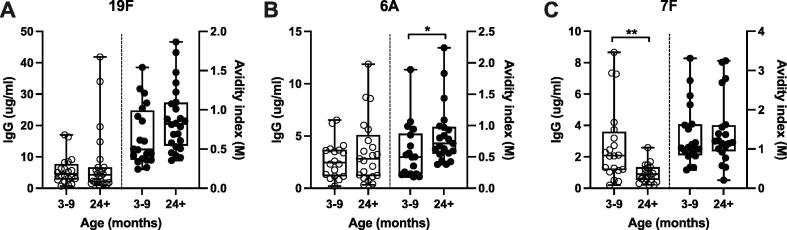
Antibody titres and avidity to *S. pneumoniae* vaccine serotypes in Malawian children. Box and whisker plots show IgG titres (ug/ml, left y-axis, empty circles) and IgG avidity index (M, right y-axis, filled circles) for children aged 3–9 months (n = 18–20) and >24 months (n = 20–24) for serotypes 19F, 6A and 7F (A-C). Box-and-whisker plots indicate medians, IQR and min/max values. Statistical analysis by unpaired Mann-Whitney *t* test, * p <0.05, ** p <0.01.

Having demonstrated that PCV13 vaccination induces high IgG titres and OPK responses in adults and that in unvaccinated adults high avidity antibodies are associated with reduced experimental colonisation, we investigated whether there was a relationship between vaccine or naturally induced antibody and colonisation in a population with high colonisation prevalence in Blantyre, Malawi [11]. We have previously shown that, amongst children under 5 years of age in this population, there is high residual carriage of vaccine serotypes in a context of high vaccine uptake [11]. We therefore utilised a serosurvey in the same population [14] to measure total IgG titres and avidity to three vaccine serotypes, two of which are commonly in circulation in Blantyre, Malawi, 19F and 6A, and one which is not commonly circulating, 7F (Fig. 5).

We found that vaccination induces an antibody response greater than the 0.35 μg/ml putative protective threshold for invasive pneumococcal disease for all three serotypes in children aged 3–9 months (GMC IgG for 19F, 4.22 μg/ml [95% CI 2.86–6.22]; 6A, 2.11 μg/ml [1.38–3.22]; 7F, 1.88 μg/ml [1.19–2.96]) [14], and that antibody responses to 19F and 6A remained above this threshold in children over 2 years old (Fig. 5A-B) (GMC IgG for 19F, 4.31 μg/ml [95% CI 2.80–6.63]; 6A, 2.38 μg/ml [1.52–3.72]) [14]. In contrast, antibody titres against 7F decreased with age (Fig. 5C) (GMC IgG 0.78 μg/ml [0.57–1.08]). This may reflect exposure to the residual carriage of 19F and 6A but limited exposure to 7F in this population [11], [14]. IgG1 and IgG2 titres were measured in these sera and followed the same patterns as IgG for each serotype (data not shown) but did not add further nuance to our results.

Avidity was stable across both age groups for all three serotypes, despite the decrease in 7F-specific IgG titres (GMAI at 3–9 months vs 2+ years for 19F, 0.58 vs 0.79, 95% CI 0.44–0.75 vs 0.65–0.97; 6A, 0.45 vs 0.76, 95% CI 0.32–0.64 vs 0.61–0.94; 7F, 1.16 vs 1.20, 95% CI 0.90–1.49 vs 0.89–1.64). Avidity was thus maintained even in the absence of exposure to antigen and was slightly higher in older children against circulating serotypes, which reached statistical significance for 6A (p = 0.025) although not 19F (p = 0.060).

**Fig. 6:**
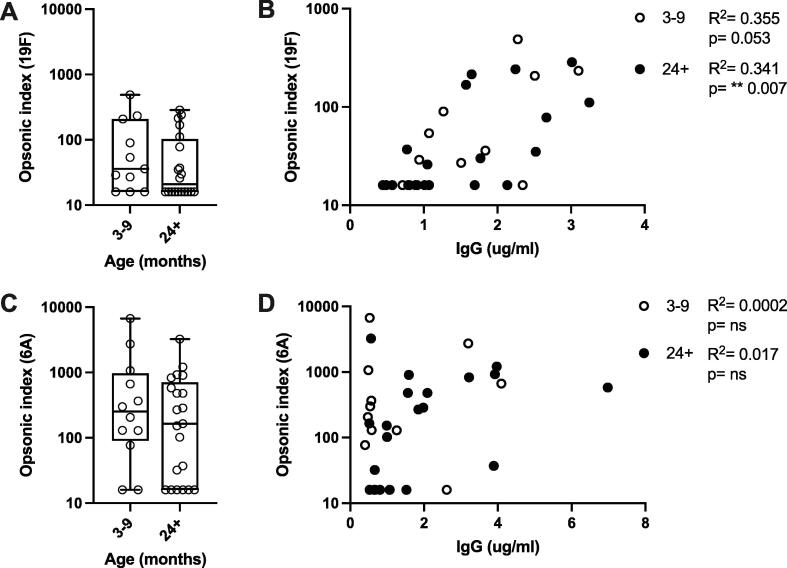
Opsonophagocytic killing responses in Malawian children to *S. pneumoniae* serotypes 19F and 6A. OI values are divided by age group (3–9 months (n = 11) or >24 months (n = 20)) for serotypes 19F (A, C) and 6A (B, D). Box-and-whisker plots indicate medians, IQR and min/max values. Scatter plots show OI vs whole IgG titre (ug/ml). R^2^ and p values shown on each plot indicate analysis via linear regression. ** p <0.01.

OPK responses to serotypes 19F and 6A were low post-vaccination and did not increase with exposure (Fig. 6A-B), and indeed, they declined (geometric mean OI at 3–9 months vs 2+ years for 19F, 55 vs 37, 95% CI 24–123 vs 22–61; 6A, 260 vs 141, 95% CI 81–838 vs 63–317). Our data also show a relationship between IgG titres and OPK responses for serotype 19F in both age groups (Fig. 6C) (3–9 months, R^2^ = 0.355, p = 0.053; 2+ years, R^2^ = 0.341, p = 0.007), but not for serotype 6A (Fig. 6D) (3–9 months, R^2^ = 0.0002, p = 0.966; 2+ years, R^2^ = 0.017, p = 0.574).

## 4 Discussion

In young UK adults, we show that PCV13 vaccination generates functional IgG1 and IgG2 antibodies and OPK activity. While we were not able to show a direct relationship between vaccine-induced functional antibody and protection against carriage, vaccination was protective against carriage and vaccinated individuals who did become experimentally colonised had a reduced colonisation density compared to controls [7]. Additionally, in a control HepA-vaccinated population we show that high avidity naturally-acquired antibody at baseline is linked to reduced likelihood of carriage after experimental challenge. However, in contrast, in a childhood population with high carriage prevalence we did not find a relationship between antibody levels, OPK activity or antibody avidity and pneumococcal colonisation at the population level.

Adults in the EHPC study had strong IgG2 responses to PCV vaccination, consistent with previous studies showing that PCV vaccination predominantly induces IgG2 responses in adults [26], [27]. As expected, IgG1 responses were more modest [26] and correlated with OPK activity, which is consistent with known functional activities of this subclass [25]. OPK responses also increased substantially after vaccination but were not linked to reduced likelihood of carriage or intensity of colonisation following experimental challenge with 6B. Although we did not find any correlation of OPK responses with avidity to serotype 6B here, others have found that high avidity antibodies better mediate opsonisation and OPK of bacteria [21], [42]. In the EHPC HepA-vaccinated control group, individuals with high baseline avidity were significantly less likely to become colonised after challenge, which suggests that naturally occurring high avidity antibodies may be important for protection against colonisation [32], [43]. We did not find this relationship following PCV13 vaccination of adults, but as very few vaccinated individuals became colonised after challenge, it was not possible to identify significant differences in their antibody responses compared to uncolonised individuals.

PCV13 vaccination generated high antibody titres against serotypes 6B and 19F in adults but the overall antibody avidity for both serotypes decreased. This is likely to represent a dilution of the higher avidity antibody seen at baseline by lower avidity antibodies induced by vaccination, which is supported by the negative correlation between avidity and IgG titre after vaccination. The generation of low avidity antibody after vaccination was unexpected, as other studies have generally found that avidity after PCV administration remains comparable to pre-vaccination levels [33] or increases [42]. Many participants in the EHPC study had antibodies from previous exposure, but none had previously received a pneumococcal vaccine [7]. Vaccination may therefore have stimulated other *de novo* B cell responses in addition to existing responses, generating low avidity antibodies prior to affinity maturation, whereas vaccination of individuals who had previously received PCV would trigger recall responses and high-affinity antibody production. The rapid waning of OPK responses in the PCV13-vaccinated individuals also suggests that a proportion of the measured OPK is IgM-driven [44], which further supports the possibility that some of these responses are primary B cell responses.

Decreases in antibody avidity have previously been seen 1 month after vaccination in infants receiving a first PCV dose but not in infants receiving a booster vaccination at the same time point [45]. Low avidity antibodies may therefore represent the initial immune response to vaccination prior to maturation of the immune response via subsequent vaccine doses or exposure [46], whereas over time lower titres of high avidity antibodies prevent colonisation [21], [43]. Previous studies proposed a protective role against carriage acquisition for memory B cells generated through natural exposure to *S. pneumoniae* [22] and we therefore speculate that locally produced high avidity antibodies may be important for the control of carriage.

Although Malawian children vaccinated with PCV13 showed robust responses to 6A, 19F and 7F VTs and had measurable antibody titres after natural re-exposure to 6A and 19F *S. pneumoniae*, they did not generate strong OPK responses. Avidity appeared to be maintained over time even in the absence of re-exposure to antigen, and previous studies have found that the infant immune system, primed by PCV vaccination and subsequent natural exposure to *S. pneumoniae*, generates high avidity antibodies [31], [45], [46]. Conversely, we measured low OPK responses in young children and suggest that OPK responses increase with age, which is consistent with other studies showing that children receiving a booster dose at 24 months rather than 12 months had significantly higher OPK responses a month after immunisation [45]. Whether the difference in correlation of IgG titre with OPK activity between 19F and 6A seen in our study reflects the relative contributions of IgM and IgG to OPK responses [47] and the ratio of IgG1 to IgG2 generated through vaccination and natural exposure remains to be determined [44].

Of note, studies have found that infants in Kenya [48] and Israel [49] colonised with certain serotypes including 6A and 19F prior to or during childhood vaccination subsequently had lower serotype-specific IgG titres after vaccination compared to children who were not colonised with that serotype. The effect of pre-vaccination colonisation on OPK and avidity is currently unknown, but a better understanding of these parameters, and particularly whether avidity is reduced or increased as a result, would be beneficial for determining the impact of PCV in high-burden settings. This may also have implications for closely related serotypes and whether colonisation with one serotype affects antibody responses to related serotypes; however, there is evidence that cross-reactive antibodies to serotypes 6A and 6B show strong OPK responses [44] and similar avidity towards each serotype [50], suggesting that cross-reactive functional antibody responses may contribute to protection against colonisation.

This study has several limitations. Firstly, serum antibody responses may only partially reflect immunity at the mucosal surface which mediates the control of pneumococcal colonisation. Nonetheless, serum responses may be indirect correlates of protection against colonisation without being mechanistically linked to protection [9], [14]. Secondly, the low frequency of *S. pneumoniae* carriage positive adults following PCV13 in the EHPC study precluded in-depth analysis of their antibody quality. Thirdly, our evaluation of functional antibody was limited by the nature of the serosurvey in Malawi as we could not follow individual children to assess whether antibody titres were linked to exposure or if antibody quality determined subsequent colonisation with *S. pneumoniae*. Lastly, determining the functional ability of IgG1 and IgG2 in these children were not possible due to low remaining volumes of sera, but further analysis of these subclasses may better elucidate the connections between subclass, avidity, and OPK after vaccination and colonisation.

In conclusion, our findings implicate the quality of antibody in the control of carriage in adults. At the population level, we did not establish a link between functional antibody (OPK activity and avidity) in a vaccinated childhood population in Malawi with a high carriage burden, but we postulate that immune maturation with age, as well as repeated exposure through carriage events, may be important determinants of protection against carriage. In the light of our data demonstrating ongoing VT carriage [11] and waning immunity in the second year of life [14], as new PCV formulations and schedules are evaluated, further measures of antibody function in the context of immune maturation will be crucial to understand the control of pneumococcal colonisation.

## Funding

This work was supported by the Bill & Melinda Gates Foundation, USA [Grant No OPP117653], and the National Institute for Health and Care Research (NIHR) [grant number 16/136/46] using UK aid from the UK Government to support global health research. RSH is NIHR Senior Investigator. MAG is funded by a Research Professorship from the NIHR (NIHR300039). The Malawi-Liverpool-Wellcome Programme is core-funded by a grant from the Wellcome Trust (206545/Z/17/Z).

The views expressed in this publication are those of the author(s) and not necessarily those of the NIHR or the UK government. The funders had no role in study design, collection, analysis, data interpretation, writing of the report or in the decision to submit the paper for publication. The corresponding author had full access to the study data and, together with the senior authors, had final responsibility for the decision to submit for publication.

## Declaration of Competing Interest

The authors declare that they have no known competing financial interests or personal relationships that could have appeared to influence the work reported in this paper.

## Data Availability

Data will be made available on request.
