# Supplementary material for: Quality of antibody responses by adults and young children to 13-valent pneumococcal conjugate vaccination and Streptococcus pneumoniae colonisation
Source: Vaccine. 2022 Nov 28;40(50):7201–10. doi: 10.1016/j.vaccine.2022.09.069 (PMC10615833; doi:10.1016/j.vaccine.2022.09.069)

**Quality of antibody responses by adults and young children to 13-valent pneumococcal conjugate vaccination and *Streptococcus pneumoniae* colonisation, Wolf et al**

**Supplementary data**

Supplementary Figure 1: Serotype 19F-specific IgG (A) and avidity (B) responses for HepA-vaccinated individuals at baseline (n=10) and PCV13-vaccinated individuals at baseline and after vaccination (n=10) as part of the EHPC model. Statistical analyses carried out by Wilcoxon signed rank tests. * p< 0.05. For change in IgG titre before and after vaccination in PCV13-vaccinated individuals, p= 0.0195; for change in avidity, p= 0.0781.


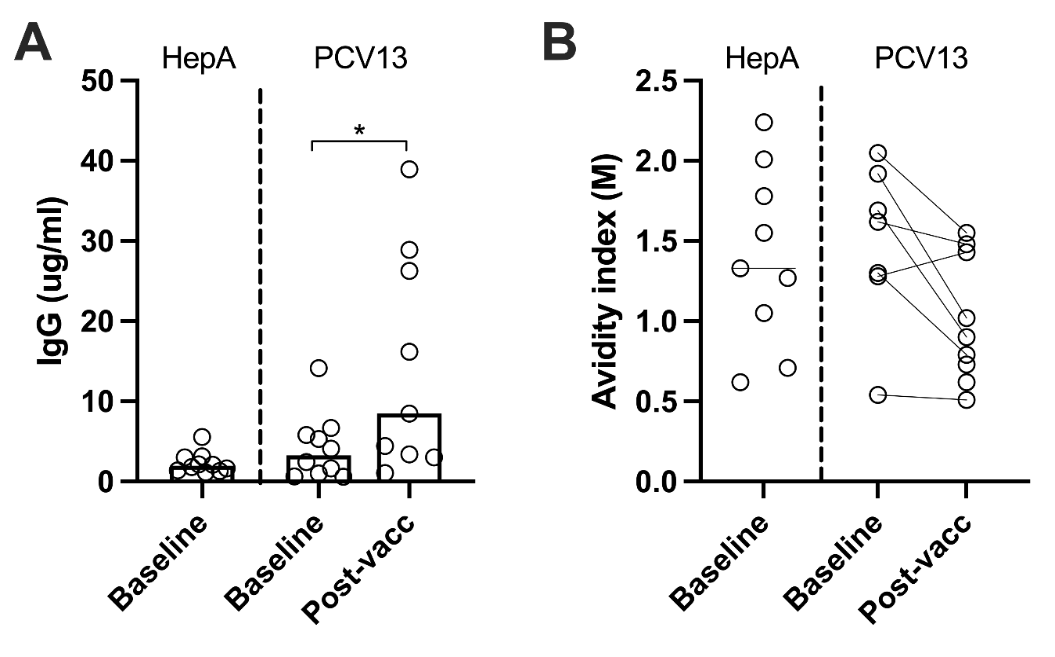

Supplement: Supplementary data 1 [file mmc1.docx]
